# *MechaSuite*: An Integrated Software for Chemical Reaction Mechanism Analysis and Microkinetic Modeling

**DOI:** 10.1021/acs.jcim.6c00861

**Published:** 2026-05-06

**Authors:** Reisel Millán, Miguel Ródenas, Alechania Misturini

**Affiliations:** † Instituto de Tecnología Química, 52735 Universitat Politècnica de València − Consejo Superior de Investigaciones Científicas (UPV-CSIC), Avenida de los Naranjos s/n, València 46022, Spain; ‡ Institut de Ciència Molecular, Universitat de València, Catedrático José Beltrán 2, Paterna 46980, Spain

## Abstract

We present *MechaSuite*, an open-source modular software suite designed to streamline the analysis of quantum-chemical reaction mechanisms. *MechaSuite* combines an intuitive data manager (*MechaData*), a molecular geometry editor (*MechaEdit*), and a microkinetic modeling engine (*MechaKinetics*). It facilitates the calculation of thermodynamic and kinetic parameters from quantum chemical outputs, the visualization and editing of molecular structures, and the simulation of complex reaction networks. This integration enables chemists to transition seamlessly from ab initio calculations to kinetic predictions in a user-friendly and efficient manner. *MechaSuite* is primarily implemented in Python with its high-performance 3D visualization engine written in C++ for optimal rendering and interactivity.

## Graphical abstract

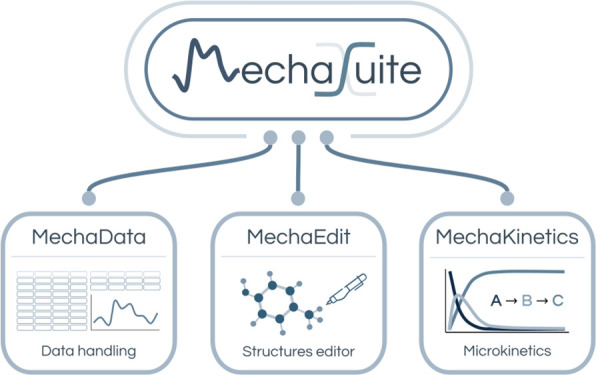

## Introduction

In the realm of chemical reactions, understanding not just *what* happens but *how fast* and *why* it happens at that rate falls under the umbrella of chemical kinetics. This field provides the fundamental tools to dissect the intricate pathways reactions take, offering crucial insights into optimizing industrial processes, designing new catalysts, and even shedding light on biological mechanisms. Understanding complex reaction mechanisms at the molecular level relies on the application of transition state theory,^1^ in which rate constants of elementary steps are related to activation free energies according to Eyring’s equation (eq 1):

$$k=\frac{\kappa k_{B}T}{h}e^{-\Delta G^{‡}/RT}$$ 1

where *k* is the rate constant, Δ*G* ^‡^ is the Gibbs energy of activation, κ is the transmission coefficient, *k*_B_ is the Boltzmann constant, *T* is the temperature, and *h* is the Planck constant.

A prerequisite for using this relation is obtaining accurate electronic energies, a step commonly accomplished with quantum chemistry methods like density functional theory (DFT)^2,3^ or post-Hartree–Fock techniques,^4^ implemented in software packages such as Gaussian,^5^ ORCA,^6^ Q-Chem,^7^ VASP,^8^ etc. While these programs provide high-quality electronic structure calculations, the downstream processing of results—especially for entire reaction mechanisms—often remains manual, fragmented, and error-prone.

For isolated elementary steps or small systems, the extraction and interpretation of quantum-chemical output (e.g., energies, geometries, and transition states) can be performed in a relatively simple manner. However, when extended to reaction networks (i.e., interconnected assemblies of elementary reactions) comprising tens or even hundreds of intermediates and transition states, the situation becomes substantially more complex.

The analysis of reaction networks requires consistent energy referencing, identification of reaction paths, and systematic comparison of competing pathways. Without specialized tools, handling these networks quickly becomes cumbersome or even intractable, as manual tracking of numerous species, their energetic relationships, and connectivity can lead to inconsistencies and incorrect mechanistic interpretation. Consequently, efficient and reliable postprocessing workflows are essential for transforming raw quantum-chemical data into chemically meaningful reaction mechanisms. Therefore, the transition from basic spreadsheet use to specialized computational tools is critical for effective data handling, thermochemical analysis, visualization, and kinetic modeling.

Over the past decade, several specialized tools have been developed to automate the extraction, analysis, and organization of computational chemistry data. *GoodVibes* ^9^ is a Python script that parses vibrational frequency outputs to compute thermodynamic corrections, offering features like lightweight entropy corrections, frequency scaling, and tabular summaries. For kinetic modeling, *AutoTST* ^10^ automates the generation of transition state structures and reaction rate constant calculations, often feeding into larger packages like *Reaction Mechanism Generator* (RMG),^11–13^ a powerful tool for rule-based generation of kinetic models for combustion and catalytic systems. *Overreact* is a command-line tool and Python library for building and analyzing homogeneous microkinetic models using ab initio calculations.^14,15^

Other notable tools include *ASE* (Atomic Simulation Environment),^16^ a versatile Python library for manipulating atomic structures and performing simulations using various backends, *ChemTraYzer,* ^17^ and *KinBot*,^18^ which specialize in the automated identification of reaction pathways and the construction of transition state guesses, *Cantera*, for chemical kinetics and transport modeling,^19^ and *NanoReactor*, for ab initio reaction discovery and network generation.^20^

Finally, CatMAP is a python package for descriptor-based microkinetic mapping of catalytic trends.^21^ Its aim is to determine the minimum set of descriptors (e.g., binding energies, etc.) that explain observable reaction rates, reducing the dimensionality of a kinetic model. It is worth noting that CatMAP is primarily designed for steady-state microkinetics rather than transient dynamics. Regarding the latter, OpenMKM is an open-source multiphysics platform developed for modeling heterogeneous catalytic reactions with a primary focus on transient kinetics and time-dependent species evolution.^22^ Another example for transient dynamics is MKMCXX, a C++-based engine that allows simulation of large reaction networks by solving a system of differential equations and includes integrated analytical tools like degree of rate control analysis.^23^ Input files can be generated by using the graphical user interface (GUI) AMSkinetics, which is part of the commercial package SCM.^24^ This nonopen-source nature represents, in our assessment, a limitation that must be taken into account.

Although a variety of computational tools are available, the majority are predominantly script-based, thereby necessitating advanced programming expertise and limiting accessibility for researchers without such background. Furthermore, these tools are largely oriented toward automation rather than postprocessing and consequently provide only restricted linear output handling without integrated support for mechanism visualization, structural editing, or spreadsheet-style management of reaction mechanisms. While certain frameworks exhibit considerable flexibility, their reliance on extensive scripting constrains their usability as they generally lack high-level interfaces tailored to reaction mechanism workflows and emphasize automated discovery over manual refinement and thermodynamic analysis. The *MechaSuite* platform addresses these limitations by providing an integrated, visual, and interactive solution specifically designed for organizing and analyzing reaction mechanisms based on quantum chemical calculations.

## Architecture and Components of *MechaSuite*

Building upon this idea, *MechaSuite* was developed as a modular platform that unifies data handling, structural visualization, and kinetic modeling (see Figure 1). Its architecture promotes an efficient workflow, enabling users to transition effortlessly among the different stages of mechanistic analysis.

**Figure 1:**
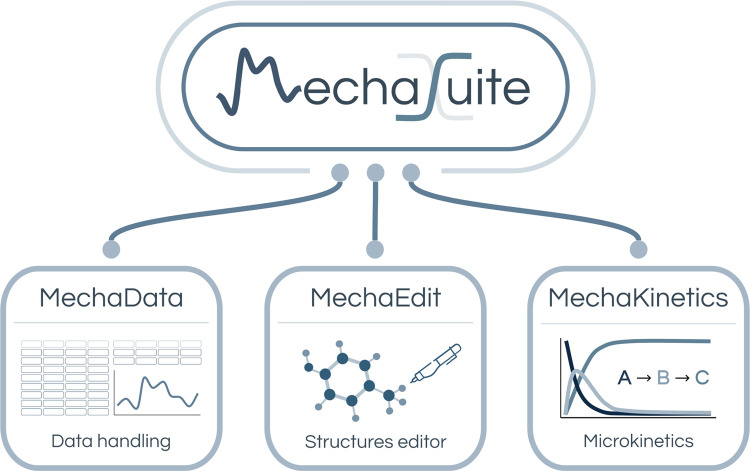
Structure of the *MechaSuite* environment, integrating *MechaData*, *MechaEdit*, and *MechaKinetics* tools.

*MechaSuite* is developed primarily in Python, leveraging its ecosystem of scientific libraries for data processing, numerical analysis, and user-interface development. In particular, the PyQt5 library^25^ is used for GUI rendering, NumPy library^26^ for numerical operations, Matplotlib^27^ for plotting energy profiles, and SciPy^28^ for numerical solving systems of differential equations. The 3D molecular visualization and editing functionality in *MechaEdit* is implemented in C++, employing an OpenGL backend^29^ and the Qt library^30^ to ensure smooth rendering, responsive user interaction, and efficient handling of complex molecular systems.

### *MechaData*: Reaction Data Manager and Thermochemical Analyzer

The first module, *MechaData*, provides a unified, spreadsheet-style environment (Figures 2 and S3a in the Supporting Information (SI)) for managing and analyzing quantum-chemical results associated with reaction mechanisms. It allows users to import and organize data from geometry optimizations, vibrational analyses, and transition-state calculations, automatically extracting structural and energetic information from computational chemistry outputs. Reaction mechanisms are arranged in a flexible, column-based layout, where each column represents a distinct reaction network and species are classified as intermediates, transition states, or reference states. An integrated geometry visualization (Figure S3d in the Supporting Information) further supports efficient inspection and validation of computed structures, offering an intuitive, GUI-based workflow that emphasizes mechanistic understanding rather than manual data handling.

**Figure 2:**
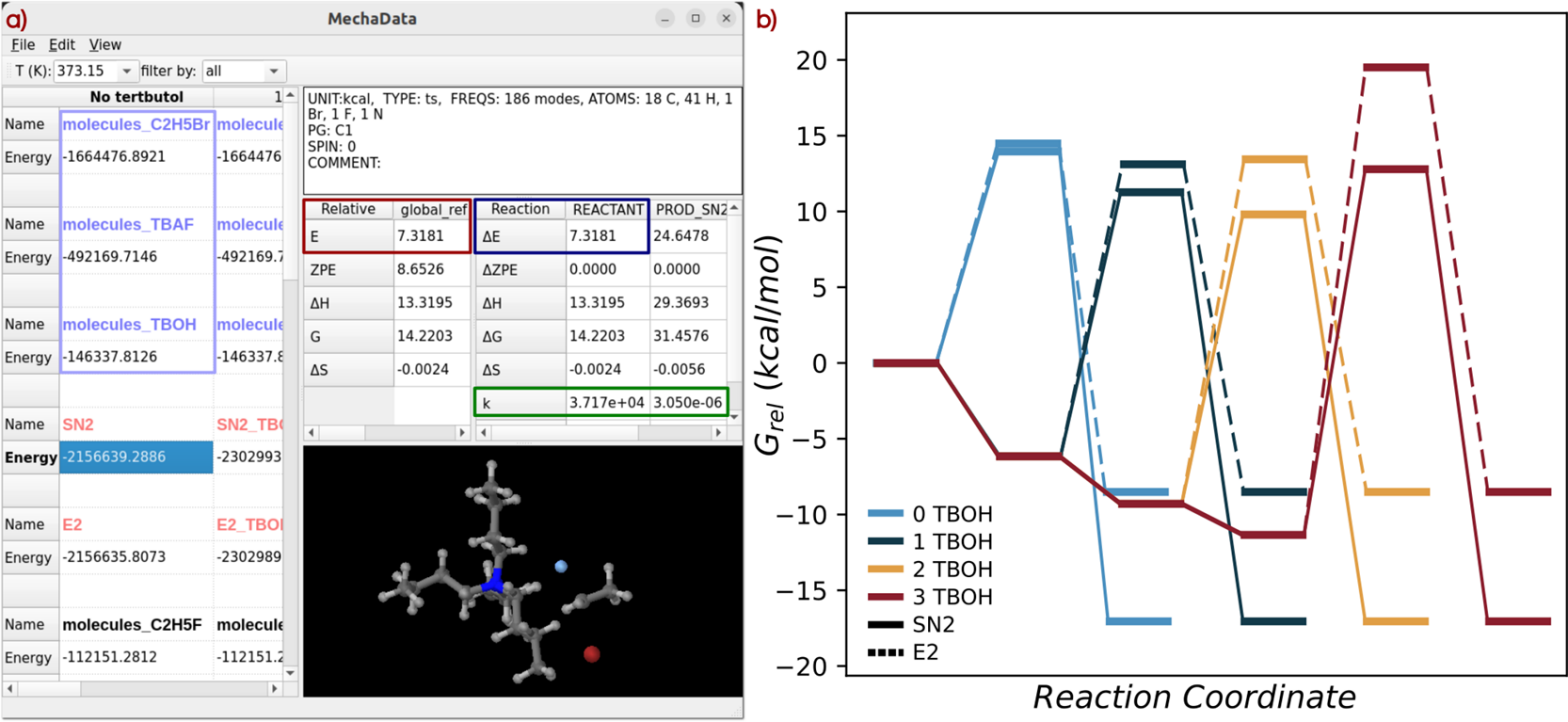
(a) Snapshot from *MechaData* showing *fluorination.json*. In the spreadsheet, structure types are color-coded, with light purple indicating reference species, light red denoting transition states, and black corresponding to minimum structures. Colored boxes indicate selected entries discussed throughout this section. (b) Free energy profiles of S_N_2 and E2 reactions for 0, 1, 2, and 3 TBOH molecules, at 373.15 K.

Another strength lies in its comprehensive thermochemical and kinetic processing capabilities. The module computes entropic contributions from vibrational frequency analyses and derives thermodynamic quantities such as enthalpies and Gibbs free energies (Figure S3b,c and Sections S1.1 and S1.2 in the Supporting Information). Rate constants are automatically evaluated from activation free energies using the transition-state theory, at the specified temperatures and with consistent unit conversions. Relative energies are also managed automatically. Users can define reference calculations from which relative energies are derived for all of the species in a mechanism. This approach ensures that intermediates can be consistently merged into a new intermediate (for practical purposes) by summing their energy contributions, maintaining coherent and reproducible energy profiles, even in complex systems with multiple reactants or fragments. These features minimize human error and provide a robust foundation for subsequent kinetic or microkinetic analyses.

*MechaData* includes an interactive plotting tool that transforms organized thermochemical data into clear visual representations. Free energy profiles along reaction coordinates can be generated directly within the interface (Figure S8b of the Supporting Information), enabling rapid assessment and comparison of competing pathways and offering a clear overview of the energetic landscape of a mechanism. The plotting system supports customization of styles, labels, and layouts and produces high-quality, exportable figures suitable for publication using the Matplotlib library.

Unlike many comparable tools, MechaData provides a fully featured GUI that requires no coding knowledge, making the platform accessible to a broader community of chemists, including experimentalists seeking mechanistic insights. At the same time, users who prefer to work programmatically can export the mechanisms in JSON, allowing for further customization or integration into computational workflows. Also, the postprocessed data can be easily exported in structured formats (CSV and xlsx files), facilitating reproducibility, data sharing, and further analysis.

While the interface remains highly visual and interactive, its design promotes transparent, reproducible workflows in mechanistic modeling (implementation theoretical background is outlined in Section S1 of the Supporting Information). A detailed description of menu functions, user actions, and energy referencing is provided in Section S4 of the Supporting Information.

### *MechaEdit*: Geometry Visualizer and Editor

*MechaEdit* provides a fully featured 3D molecular viewer and structure editor (Figure S3e in the Supporting Information). Although basic visualization capabilities are integrated into *MechaData*, *MechaEdit* is specifically designed for a detailed structural analysis and editing. Users can interactively manipulate atom positions and atomic types and adjust bond lengths and angles in an intuitive graphical environment with real-time visual feedback of how the geometrical parameters change. The editor supports common manipulation operations such as copy, paste, delete, and multistep undo/redo, allowing flexible and error-tolerant editing. It supports standard molecular file formats, such as XYZ, POSCAR (VASP input with geometry information), OUTCAR (VASP output containing vibrational frequencies), and CIF to facilitate interoperability with a wide range of quantum chemistry software. Its flexible selection system enables atoms or fragments to be manipulated based on the element type, atom classification, bonding environment, spatial criteria (e.g., spherical regions), or atom indices. Additional options include the analysis of vibrational modes and trajectories resulting from molecular dynamics simulations. The editor is particularly useful for building model systems and preparing structures for reoptimization based on previously converged intermediates. A detailed description of *MechaEdit* capabilities is provided in Section S5 of the Supporting Information.

### *MechaKinetics*: Microkinetic Modeling and Solver

The final module, *MechaKinetics*, is a microkinetic modeling engine that employs rate constants and thermodynamic parameters calculated in *MechaData* to solve reaction networks. It incorporates a differential equation solver capable of predicting time-dependent concentration profiles of intermediates (see the theoretical foundation in Section S1.3 of the Supporting Information). Reaction networks and operating conditions can be defined directly via the structured input files.

A major advantage of solving the system of differential equations that defines the reaction network is the possibility of calculating apparent activation energies and reaction orders for arbitrary mechanisms by running several simulations at different temperatures or reactant concentrations. Further details can be found in Section S6 of the Supporting Information.

## Examples and Discussion

A typical workflow begins with importing quantum-chemical output files into *MechaData*. Then, users can compute thermodynamic and kinetic properties at a given temperature, validate geometries using the built-in or advanced visualizer (*MechaEdit*), and model reaction networks in *MechaKinetics*. This end-to-end workflow enables researchers to derive mechanistic insight from first-principles calculations with minimal manual intervention and compare theoretical microkinetic results directly with experimental data.

To demonstrate *MechaSuite'*s capabilities, we present a hypothetical first-order reaction (Section S7 of the Supporting Information) and a competition between S_N_2 and E2 mechanisms from the literature. These examples illustrate how *MechaSuite* facilitates the extraction of kinetic parameters from quantum data, the construction and visualization of free energy profiles, and the simulation of full kinetic behavior under experimental conditions. Furthermore, *MechaSuite* has already been employed in DFT-based studies of heterogeneous catalysts, encompassing both structural and kinetic aspects.^31^

To illustrate the complete *MechaData* workflow and, in particular, how DFT-based microkinetic analysis can be employed to investigate competing reaction pathways, we chose the study of Lisboa and Pliego Jr.,^32^ in which the influence of microsolvation on the selectivity between S_N_2 and E2 mechanisms was assessed. The reaction involves the nucleophilic fluorination of ethyl bromide with tetrabutylammonium fluoride (TBAF), and the effect of tert-butanol (TBOH) molecules as microsolvating species is examined. In this system, the desired S_N_2 substitution (reaction 2) competes with the E2 elimination of ethyl bromide by TBAF, yielding ethylene and HF (reaction 3). To ensure consistency with the reference study, we reused the molecular structures provided by the authors and recalculated all electronic energies and vibrational frequencies using the same computational methodology and software, except for the implicit solvent model, which was omitted. The ORCA-optimized minimum-energy and transition-state geometries, together with their corresponding vibrational frequencies, are available in the directory *examples/example_2* of the source code. Moreover, a complete JSON input file *fluorination.json* is included to enable full reproduction of the results presented here.

$${\mathrm{CH}}_{3}{\mathrm{CH}}_{2}\mathrm{Br}+\mathrm{TBAF}\overset{S_{N}2}{\underset{nT\mathrm{BOH}}{⇌}}{\mathrm{CH}}_{3}{\mathrm{CH}}_{2}F+\mathrm{TBABr}$$ R1

$${\mathrm{CH}}_{3}{\mathrm{CH}}_{2}\mathrm{Br}+\mathrm{TBAF}\overset{E2}{\underset{n\mathrm{TBOH}}{⇌}}C_{2}H_{4}+\mathrm{HF}+\mathrm{TBABr}$$ R2

Upon opening the input file (*fluorination.json*) with *MechaData*, a four-column spreadsheet is seen (Figure 2a). These columns contain the S_N_2 and E2 mechanisms for systems including up to 3 TBOH molecules. Each row entry corresponds to one of the mechanism transition states or intermediates (geometry-optimized structures of the local minima).

The calculated relative energy of each intermediate can be accessed by just clicking on the intermediate cell and is shown on the relative energy panel (denoted by the dark red box in Figure 2a). For instance, the relative energy of the S_N_2 transition state is 7.3 kcal/mol. In addition, reaction energies and activation energies of an arbitrary intermediate can also be calculated with respect to any other intermediate, as long as they have the same number of atomic types or the same reference. In this example, the activation energy of the S_N_2 transition state with respect to the intermediate *REACTANT* is also 7.3 kcal/mol (framed in navy blue in Figure 2a), as the relative energy of *REACTANT* is zero kcal/mol. This is because *REACTANT* is formed by merging two reference species (see further details about energy referencing in Section S4.1.6 in the Supporting Information).

For a better comparison with experimental data, it is also possible to automatically calculate thermodynamic quantities such as enthalpy, entropy, and free energies of activation and reaction. These quantities can be obtained by simply selecting the desired intermediates followed by right-clicking and choosing the option *Thermochemical Analysis* (Figure S5c of the Supporting Information) and specifying temperature, pressure, and volume (see Section S4 in the Supporting Information for further details). This analysis also automatically computes the rate constants for every free energy of activation. As highlighted by the green box in Figure 2a, the rate constant associated with the S_N_2 transition state at 373.15 K is 3.717 × 10^4^ s^–1^ for the direct step, and 3.05 × 10^–6^ s^–1^ for the reverse step.

Once the relative electronic energies, the free energies, or both have been calculated for all of the desired intermediates, they can be directly compared in a single energy profile. *MechaData* supports the creation of multiple energy profiles and simultaneous visualizations on the same interface (Figure S8b in the Supporting Information). Then, a fully customized and publication-ready figure can be exported as shown in Figure 2b, which summarizes the free-energy profiles of the competing reactions as a function of the number of explicit TBOH molecules. Increasing microsolvation of the transition states by TBOH leads to a systematic increase in the activation free energy difference between the S_N_2 and E2 pathways, from 0.50 kcal/mol with no TBOH molecules to 6.47 kcal/mol with three, with intermediate values of 1.97 and 3.68 kcal/mol for one and two molecules, respectively.

The activation free energy differences obtained follow the trend reported in the original study, despite the absence of solvent effects, confirming the reliability of the methodology and of the workflow implemented in *MechaData*. Furthermore, because ORCA provides free energies directly from frequency calculations, the absolute thermal and entropic corrections of each intermediate (and consequently their absolute free energy) can be directly compared to those calculated in *MechaData*, confirming exact agreement with the values reported by ORCA at the specified temperature.

Finally, a more experimentally relevant analysis can be carried out using the *MechaKinetics* module, based on the information derived from the free energy profiles. An input file containing the rate constants for all elementary steps included in the profile at each specified temperature can be exported directly from the plotting interface (see Section S6 of the Supporting Information for further details). This input is then used by the *MechaKinetics* module to solve the corresponding system of ordinary differential equations and generate concentration profiles (Figure 3).

**Figure 3:**
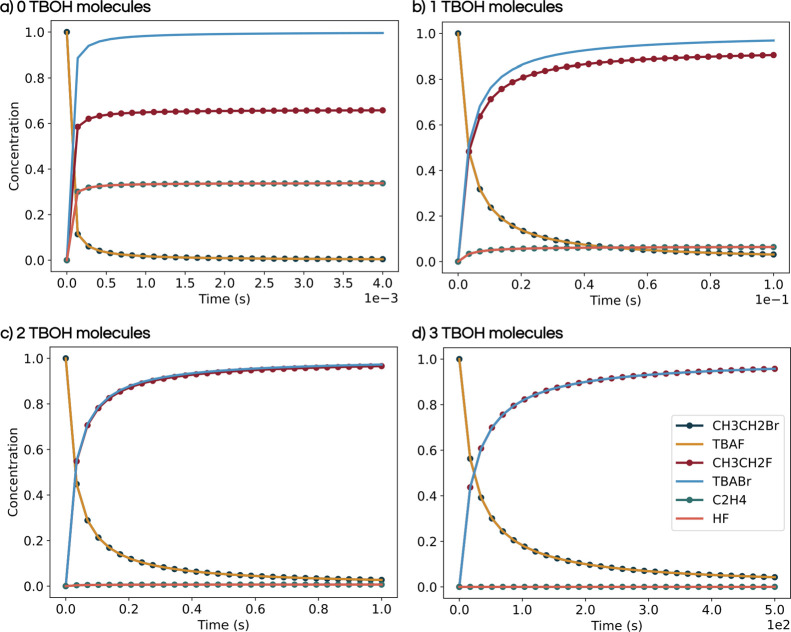
Microkinetic models of the competing S_N_2 and E2 reactions at 373.15 K, with (a) 0, (b) 1, (c) 2, and (d) 3 TBOH molecules.

Once the system reaches equilibrium, the product concentration ratio can be used to compute the reaction selectivity, as defined in eq 4.

$$S_{{\mathrm{CH}}_{3}{\mathrm{CH}}_{2}F/C_{2}H_{4}}=\frac{\left[{\mathrm{CH}}_{3}{\mathrm{CH}}_{2}F\right]}{\left[C_{2}H_{4}]+[{\mathrm{CH}}_{3}{\mathrm{CH}}_{2}F\right]}$$ 4

Figure 3 collects four microkinetic simulations performed at 373.15 K, corresponding to systems without TBOH or containing up to 3 TBOH molecules. The calculated selectivity shows a systematic increase from 65% in the absence of TBOH to 90, 96, and 99% with 1, 2, and 4 TBOH molecules, respectively, supporting the hypothesis that tert-butanol significantly enhances the selectivity toward the S_N_2 product. This trend is consistent with the findings of the original study,^32^ which employed a simpler analytical approach that, unlike microkinetic analysis, may be hard to apply to more complex systems. Finally, these results were validated through comparison with the well-established *OpenMKM* software. The corresponding discussion is included in Section S2 of Supporting Information.

## Conclusions

In summary, *MechaData* combines data structuring, thermochemical analysis, visualization, and plotting into a single GUI application. It complements existing computational chemistry tools by focusing on the downstream stages of mechanism curation, interpretation, and kinetic modeling, bridging the gap between raw electronic structure outputs and actionable chemical insights.

*MechaSuite* offers a comprehensive integrated platform for chemists studying reaction mechanisms. By combining data management, molecular visualization, and kinetic simulation, it streamlines the analysis workflow from quantum chemical results to mechanistic interpretation. Its modular design and hybrid Python/C++ implementation make it both accessible and performant. Although beyond the scope of this study, all *MechaSuite* tools can be readily applied to support chemical education. Future updates will expand compatibility with additional quantum chemistry packages, include features for automated mechanism generation, and implement advanced functionalities to the geometry viewer and editor. Furthermore, we will incorporate an implementation of kinetic Monte Carlo^33,34^ and support for reactions involving change of spin states through the nonadiabatic transition state theory (NA-TST).^35^

## Supplementary Material



## Data Availability

*MechaSuite* is available at https://github.com/rm-compchem/mechasuite. The official documentation can be found on https://mechasuite.readthedocs.io/en/latest/.

## Notes

Brief description of the fundamental principles implemented in the software, comparison of microkinetics results from *OpenMKM* and *MechaKinetics* packages, installation instructions, description of the functionalities available in *MechaSuite* graphical user interfaces, a description of *MechaKinetics* input file and an introductory example (PDF)
