# Supplementary material for: MechaSuite: An Integrated Software for Chemical Reaction Mechanism Analysis and Microkinetic Modeling
Source: J Chem Inf Model. 2026 May 6;66(10):5602–8. doi: 10.1021/acs.jcim.6c00861 (PMC13213833; doi:10.1021/acs.jcim.6c00861)
Supplement: Supplementary file 1 [file ci6c00861_si_001.pdf]

Supporting Information: *MechaSuite*: An Integrated Software for Chemical Reaction Mechanism Analysis and Microkinetic Modeling

Reisel Millán,*,† Miguel Ródenas,† and Alechania Misturini‡

†*Instituto de Tecnología Química, Universitat Politècnica de València – Consejo Superior de Investigaciones Científicas (UPV-CSIC), Avenida de los Naranjos s/n, València 46022, Spain*

‡*Institut de Ciència Molecular, Universitat de València, Catedrático José Beltrán 2, Paterna, 46980 Spain*

E-mail: reimilca@itq.upv.es

S1 Fundamentals of Kinetics

At its core, chemical kinetics seeks to elucidate the rates and mechanisms of chemical transformations. This involves exploring how reactant molecules convert into product molecules, often passing through transient, high-energy states. The following sections provide a summary of the theory behind the workflow of *MechaSuite* for the analysis of reactions mechanisms. A thorough description of the underlying formalism presented here can be found elsewhere.[S1–S3]

S1.1 Transition State Theory and Eyring Equation

One of the cornerstone theories in chemical kinetics is the Transition State Theory (TST), proposed independently by Eyring, Polanyi, and Evans in the 1930s.[S4–S6] TST provides a framework for understanding reaction rates by focusing on the properties of a special, short-lived species called transition state (TS) or activated complex.

Imagine a reaction as molecules traversing a potential energy surface (Figure S1a). Reactants reside in one valley, products in another. The transition state represents the highest energy point along the lowest energy pathway connecting reactants to products, the "saddle point" on this surface. It is a configuration where bonds are simultaneously breaking and forming (Figure S1b). TST assumes that once molecules reach this transition state, they inevitably proceed to form products.

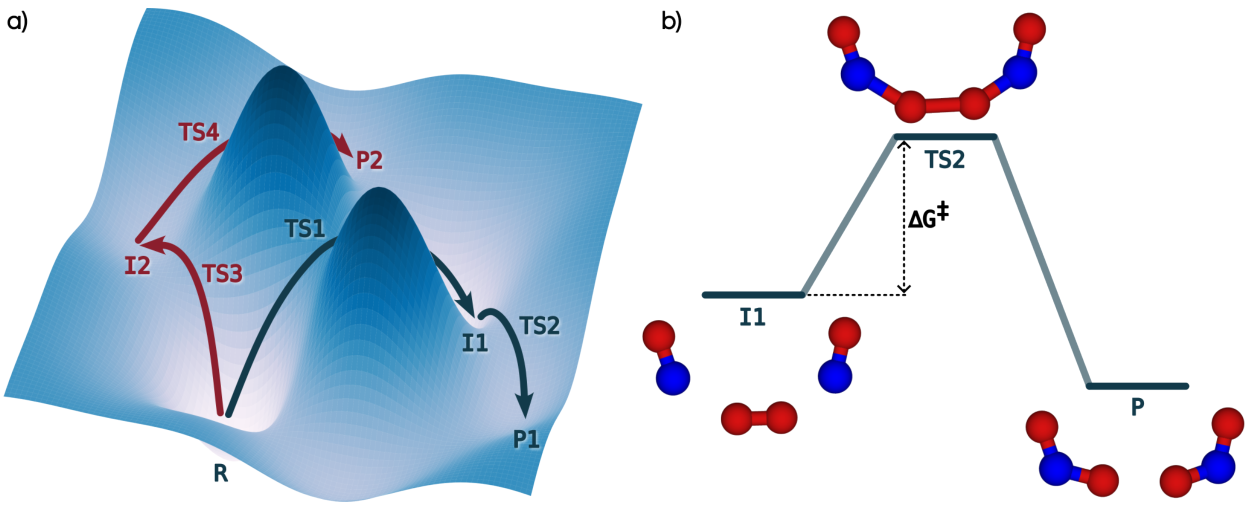

Figure S1: a) Complex potential energy landscape illustrating multiple reaction pathways, with reagents (R), local minima intermediates (I) and transition state (TS) structures indicated. b) Schematic representation of a free energy profile, illustrating the free energy of activation $\Delta G^{\ddagger}$ of the reaction.

To achieve the activated complex, reactants must surmount the free energy barrier ($\Delta G^{\ddagger}$, Figure S1b) which is a crucial thermodynamic quantity that encapsulates both enthalpy and entropy changes associated with forming the transition state. The Eyring equation (Equation S1) highlights the exponential dependence of the rate constant k on the free energy of activation, emphasizing that even small changes in $\Delta G^{\ddagger}$ can lead to significant changes in reaction rates.

$$k = \frac{\kappa k_B T}{h} e^{-\frac{\Delta G^{\ddagger}}{RT}} \tag{S1}$$

S1.2 Vibrational Analysis and Free Energy

To apply TST, we first need to determine $\Delta G^{\ddagger}$. This involves computationally characterizing the minima and transition states by determining their electronic energies and associated vibrational frequencies with quantum chemical calculations. The key distinction for the transition state is the presence of one unique imaginary vibrational frequency that corresponds to the reaction coordinate, the specific motion that leads from the transition

state to products, or back to reactants.

The vibrational frequencies obtained from quantum chemical calculations are used to evaluate all contributions to the Gibbs free energy (G), including the zero-point vibrational energy (E_{ZPV}) and the vibrational components of enthalpy (H_{vib}), entropy (S_{vib}), and, consequently, the vibrational contribution to the free energy (G_{vib}). The Gibbs free energy of activation, $\Delta G^{\ddagger}$, can be expressed as:

$$\Delta G^{\ddagger} = \Delta H^{\ddagger} - T\Delta S^{\ddagger} \tag{S2}$$

where $\Delta H^{\ddagger}$ and $\Delta S^{\ddagger}$ denote the enthalpy and entropy of activation, respectively. These quantities can be derived from the electronic energies of the reactants and the transition state, corrected for zero-point vibrational energy and including thermal contributions from translational, rotational, and vibrational motions. The total Gibbs free energy for a species is thus given by:

$$G = E_{elect} + E_{ZPV} + E_{vib} + E_{rot} + E_{trans} + RT - T(S_{vib} + S_{rot} + S_{trans} + S_{elect}) \tag{S3}$$

where E_{elect} and S_{elect} is the electronic energy and entropy, E_{vib} the vibrational energy, E_{rot} and S_{rot} are the rotational energy and entropy, E_{trans} and S_{trans} are the translational energy and entropy, T is the temperature, and R is the ideal gas constant.

By performing this analysis, it becomes possible to determine the rate constant for each elementary step within a reaction mechanism. Each step typically comprises an optimized reactant, transition state, and product. The combination of all such elementary reactions forms the reaction network, which collectively describes the full mechanistic landscape of the chemical system (Figure S1a).

S1.3 Reaction Networks and Solving Differential Equations

Real-world chemical processes rarely involve a single, isolated reaction. Instead, they typically proceed through a sequence of elementary steps that collectively form complex reaction networks (Figure S1a). Such networks may include parallel and consecutive reactions, reversible transformations, and even autocatalytic pathways. To understand and predict the behavior of these systems, it is necessary to describe how the concentrations of reactants, intermediates, and products evolve over time. This is achieved by formulating a set of ordinary differential equations (ODEs) for each species in the network. The detailed mechanistic description is referred to as microkinetic modeling, in which the rate of each elementary step in a reaction mechanism is explicitly considered at the molecular or atomic level. In this approach, no initial assumptions are made regarding the rate-determining elementary steps.[S7]

Let us consider a simple elementary reaction, which can be represented as:

$$a\ \mathrm{A} + b\ \mathrm{B} \underset{k_{1,r}}{\overset{k_{1,f}}{\rightleftharpoons}} c_1\ \mathrm{C} \tag{R1}$$

where a, b and c_1 are the stoichiometric coefficients, and $k_{1,f}$ and $k_{1,r}$ are the forward and reverse rate constants. The rate of consumption of A or B and the formation of C defines the reaction rate of the step 1 (r_1), being expressed as:

$$r_1 \equiv -\frac{1}{a}\frac{d[A]}{dt} = -\frac{1}{b}\frac{d[B]}{dt} = \frac{1}{c_1}\frac{d[C]}{dt} \tag{S4}$$

where $[A]$, $[B]$ and $[C]$ are the concentrations of the species involved. The reaction rate is proportional to such concentrations:

$$r_1 = k_{1,f}[A]^a[B]^b - k_{1,r}[C]^{c_1} \tag{S5}$$

In a complex reaction network, the rate of change in the concentration of each species depends

on the rates of all reactions in which that species participates. Therefore, if an intermediate X participates in multiple elementary steps, its temporal evolution, $d[X]/dt$, is given by the sum of all formation rates minus the sum of all consumption rates involving X.

If we add a second elementary step to the above example, such as

$$c_2\ \mathrm{C} + d\ \mathrm{D} \underset{k_{2,r}}{\overset{k_{2,f}}{\rightleftharpoons}} g\ \mathrm{G} \tag{R2}$$

with reaction rate

$$r_2 = k_{2,f}[C]^{c_2}[D]^d - k_{2,r}[G]^g \tag{S6}$$

then, the rate of change in the concentration of C would be

$$\frac{d[C]}{dt} = c_1 r_1 - c_2 r_2 \tag{S7}$$

Thus, for an entire reaction network, one can define a system of coupled differential equations, where each equation describes the rate of change of concentration for a particular species (A, B, C, D, G, etc.), such as

$$\begin{aligned}
\frac{d[A]}{dt} &= a\left(-k_{1,f}[A]^a[B]^b\ +\ k_{1,r}[C]^{c_1}\right)\\
\frac{d[B]}{dt} &= b\left(-k_{1,f}[A]^a[B]^b\ +\ k_{1,r}[C]^{c_1}\right)\\
\frac{d[C]}{dt} &= c_1\left(k_{1,f}[A]^a[B]^b\ -\ k_{1,r}[C]^{c_1}\right)\ +\ c_2\left(-k_{2,f}[C]^{c_2}[D]^d\ +\ k_{2,r}[G]^g\right)\\
\frac{d[D]}{dt} &= d\left(-k_{2,f}[C]^{c_2}[D]^d\ +\ k_{2,r}[G]^g\right)\\
\frac{d[G]}{dt} &= g\left(k_{2,f}[C]^{c_2}[D]^d\ -\ k_{2,r}[G]^g\right)
\end{aligned} \tag{S8}$$

Solving this system of ODEs yields the concentration profiles of all species as functions of time. Analytical solutions are feasible only for very simple reaction schemes. For more realistic and larger systems, numerical integration methods are required. These computational algorithms approximate the time evolution of concentrations by incrementally updating their

values based on instantaneous reaction rates. Modern software packages and programming environments equipped with ODE solvers such as MATLAB,[S8] Python (SciPy), or specialized kinetic modeling tools are therefore indispensable for simulating and analyzing the kinetics of complex reaction networks. Through such simulations, chemists can predict reaction outcomes, identify rate-determining steps, and optimize conditions to favor the formation of desired products.

To this end, *MechaKinetics* automates the construction of the system of ordinary differential equations directly from the elementary reaction steps defined by the user in a simple input file. The resulting equations are solved numerically, and the corresponding time-dependent concentration profiles are generated and visualized automatically. This approach eliminates the need for high-level programming while providing a user-friendly environment for simulating and analyzing complex reaction networks.

S2 Nucleophilic fluorination. Comparing *MechaKinetics* with *OpenMKM*

In this section, we compare the results of the fluorination reaction with the open source microkinetics software *OpenMKM*. To that end, *MechaSuite* is very convenient because it provides directly the activation energies and the pre-exponential factor requested by *OpenMKM*. The input of *OpenMKM* are different, two files are required reactor.yaml and thermo.yaml. The first contains general reactor and simulation settings, while the second defines more concretely the reaction network. In the directory *examples/example_2* in the source code, there is a subfolder named “comparison_openmkm” which contains the input files created with the reaction network information calculated from the *MechaData* interface. The example directory contains a python script “compare.py” to generate Figure S2. Because *OpenMKM* simulations and *MechaKinetics* use mean-field approximation,[S9] both simulations can be compared directly. In addition, for *OpenMKM* we have assumed ideal

gas behavior and a batch model reactor, which is what *MechaKinetics* currently supports. Other reactor models will be included in a future release. More information on *OpenMKM* input structure can be found in the literature.[S9]

Figure S2 shows the concentration profiles of the species involved in the fluorination reaction using *MechaKinetics* (top) and *OpenMKM* (bottom).

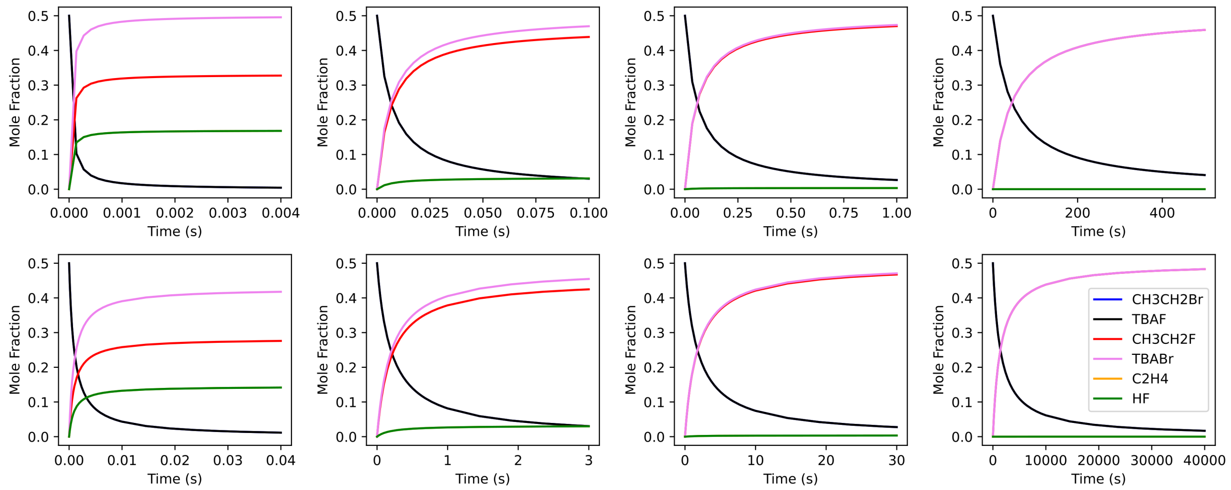

Figure S2: Comparison of the concentration profiles of microkinetics simulations with (from left to right) 0 to 4 TBOH molecules using *MechaKinetics* (top row) and *OpenMKM* (bottom row). Label CH3CH2Br always overlaps with TBAF, and C2H4 always overlaps with HF.

Both software exhibit matching concentration evolutions and reach similar equilibrium points, leading to equivalent results. We calculated the selectivities using the final concentrations from *OpenMKM* and *MechaKinetics* simulations, as shown in Table S1.

Table S1: Summary of calculated molar fractions and selectivities based on steady-state concentration profiles obtained via *OpenMKM* and *MechaKinetics* simulations.

	OpenMKM			***MechaKinetics***		
system	$\chi_{CH_3CH_2F}$	$\chi_{C_2H_4}$	% selectivity	$\chi_{CH_3CH_2F}$	$\chi_{C_2H_4}$	% selectivity
0 TBOH	0.282	0.145	66.0	0.327	0.168	66.1
1 TBOH	0.448	0.032	93.3	0.438	0.030	93.4
2 TBOH	0.474	0.003	99.2	0.470	0.003	99.3
3 TBOH	0.483	0.000	99.9	0.459	0.000	100

S3 Setting up *MechaSuite*

S3.1 Building and installing

The source code of *MechaSuite* can be found on github, and the official documentation on the Read the Docs website. The root directory contains the conda recipe for building and installing the package. As *MechaSuite* is distributed as a conda package, it can be used across Windows, macOS, and Linux systems.

With miniconda already installed, *MechaSuite* can be build and installed using the following commands:

```
# Building MechaSuite
$ conda activate base
$ conda install conda-build
$ git clone git@github.com:rm-compchem/mechasuite.git
$ cd mechasuite
$ conda-build conda-recipe -c conda-forge
# Create a conda environment called 'ms' and install MechaSuite
$ conda create -n ms
$ conda activate ms
$ conda install mechasuite --use-local
```

If you are only interested in using the Python-based modules, *mechadata.py* and *mechakinetics.py*, execute the following commands on a Linux operating system:

```
$ git clone git@github.com:rm-compchem/mechasuite.git
$ cd mechasuite
$ pip install -r requirements.txt
$ pip install .
```

Users working on Windows are encouraged to rely on Linux-compatible environments, such as Linux on Windows via the Windows Subsystem for Linux (WSL, supported in Windows 10/11), or by installing software like Cygwin.

S3.2 Using *MechaSuite* modules

If you have installed *MechaSuite* as a conda package, always activate the `ms` environment first:

```
$ conda activate ms
```

Then, *MechaData* graphical user interface (GUI) can be open by:

```
$ mechadata.py
# or opening directly a reaction mechanism from JSON file
$ mechadata.py ${MS}/mechasuite/examples/example_2/fluorination.json
```

Here, `${MS}` denotes the path to downloaded source code of *MechaSuite*. Likewise, *MechaEdit* GUI can be open by typing the following command:

```
$ mechaedit
# or opening directly a geometry file (CIF, XYZ, POSCAR or OUTCAR)
$ mechaedit ${MS}/mechasuite/examples/example_2/SN2/SN2-TBAF.xyz
```

For *MechaKinetics* usage, also indicate the JSON with the reaction mechanism:

```
$ mechakinetics.py ${MS}/mechasuite/examples/example_1/rn.json
```

S3.3 Preprocessing scripts

Importing individual calculations to an already created mechanism can be done by providing the corresponding calculation directory within the GUI. However, some preprocessing is convenient to avoid errors in trying to determine the format of the output of such calculations. To that end, we provide sample scripts that create a file named *.data* inside each

calculation directory, containing information about how *mechadata.py* should read the files in the directory. Each line in the *.data* file represents a configuration entry, specified as a *tag value* pair. The following example shows that the QM program is VASP, the file that contains the electronic energy is named OSZICAR, the file with the geometry is called CONTCAR, the energy unit is eV, the system multiplicity is singlet, the type of calculation is an optimization rather than a transition state search, and that is a periodic calculation. Other possible values are provided after the # symbol.

```
program:      vasp    # gaussian, orca
energy_file:  OSZICAR # gaussian or orca output file name or even just numerical value
struct_file:  POSCAR  # any other xyz file
unit:         eV      # kcal, kJ, Ha
spin:         0       # 1, 2, etc
tp:           min     # ts, ref
pg:           solid   # C1, Cs, C2, C2v, C3v, C2h, Coov, D2h, D3h, D5h, Dooh, d3d, Td, Oh
```

S4 *MechaData* module

MechaData distinguishes itself through several design and functional advantages. Its key strengths include:

- **Spreadsheet-like interface:** The column-based layout allows users to organize entire mechanisms, with each column representing a reaction mechanism (Figure S3a). This design offers a user-friendly interface that facilitates the analysis of reaction pathways, allowing users to focus their effort not on deciphering raw data, but on interpreting the underlying chemical processes and gaining meaningful mechanistic insight.

- **Automatic reference energy calculations:** The reliable generation and comparison of reaction pathways depend on the precise calculation of relative energies based on appropriate reference species (Figure S3b), a procedure that is often laborious and susceptible to human error. One of *MechaData*'s most distinctive features is its ability to automatically compute relative energies for all intermediates, aligning them to user-defined reference states. This reduces manual effort and ensures consistency across large datasets.

- **Built-in thermochemical tools:** Vibrational frequency data can be scaled, edited, and directly used to calculate entropies, enthalpies, and Gibbs free energies. The platform seamlessly supports post-processing of this data, its visualization via integrated plotting tools, and its application in microkinetic modeling, enabling users to transition effortlessly from thermochemical calculations to kinetic analysis.

- **Integrated visualization:** As another unique feature of *MechaData*, molecular structures can be visualized directly within the interface (Figure S3d) or in the advanced *MechaEdit* module. This functionality enables users to easily inspect the system states at each step of the reaction pathway without the need to manage multiple geometry files or relying on external visualization packages. Such accessibility provides an in-

tuitive, researcher-oriented experience that prioritizes the mechanistic understanding over manual file handling.

- **No programming required:** Unlike many comparable tools, *MechaData* provides a fully featured graphical user interface (GUI, Figure S3a-d), requiring no coding knowledge and making the platform accessible to a broader community of chemists, including experimentalists seeking mechanistic insights. At the same time, users who prefer to work programmatically can export the mechanisms in JSON, allowing further customization or integration into computational workflows. Also, the post-processed data can be easily exported in structured formats (csv and xlsx files), facilitating reproducibility, data sharing and further analysis.

- **Direct plotting of free energy profiles:** A key innovation of *MechaData* is its ability to generate free energy diagrams directly from the organized data, without the need for external plotting tools. This enables both reproducibility and customization as the diagrams can be visualized and readily adjusted (including style, formatting, labels and layout) before being exported as high-quality images using the Matplotlib python library.

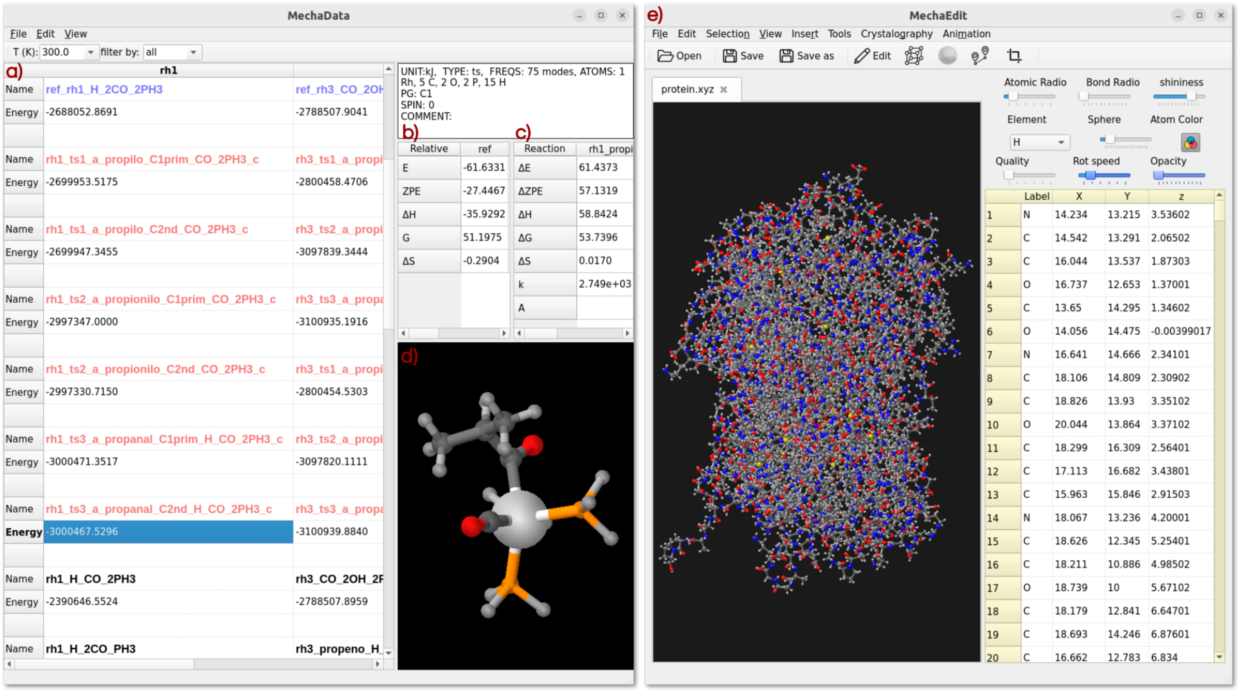

Figure S3: *MechaData* interface. a) Main spreadsheet for organizing reaction mechanisms. b) Relative energy panel and c) reaction energy panel. d) Embedded visualizer for molecular structures. e) *MechaEdit* interface.

S4.1 User interface and functionality

The GUI of *MechaData* is designed to facilitate the setup, execution, and analysis of reaction network simulations through an intuitive, user-centric layout. It combines spreadsheet-style controls with interactive visual tools, while the menu structure is organized into logical categories for data management, visualization, plotting, and mechanistic editing. This section provides an overview of the main components of the GUI and describes the functionality of the available menus.

S4.1.1 File Menu (Figure S4a)

- **(1) Import Mechanism From Folder:** Loads a reaction mechanism from a directory containing several folders (one per calculation) with quantum chemical output files. *MechaSuite* automatically extracts relevant thermochemical and structural data for

each intermediate and transition state. It is advisable to run the preprocessing scripts first, to ensure the QM software and filenames are set properly.

- **(2) Import Mechanism From File:** Loads a reaction mechanism from a JSON file to the selected mechanism (column).
- **(3/4) Save / Save As:** Saves the current workspace as a JSON file, including all mechanisms, structures, references, and calculated data. Useful for preserving project state.
- **(5) Export to Excel:** Exports the reaction data sheet to an Excel file for external analysis or reporting.
- **(7-9) Open / Close:** Opens an existing mechanism project or closes the current workspace.

S4.1.2 Edit Menu (Figure S4b)

- **(10) New Mechanism:** Creates a new reaction mechanism column within the workspace. Each column represents an independent mechanism and serves as a container for intermediates, transition states, and references.

S4.1.3 View Menu (Figure S4c)

- **(11) Plot:** Generates plots of energy profiles or kinetic trends based on the currently selected mechanism and data. Users can visualize free energy diagrams, reaction coordinates, or custom thermodynamic plots.

S4.1.4 Right-Click Options on Columns (Mechanism-Level Controls, Figure S5a)

- **(14) New Item:** Adds a new row to the current mechanism for a structure, intermediate, or transition state.

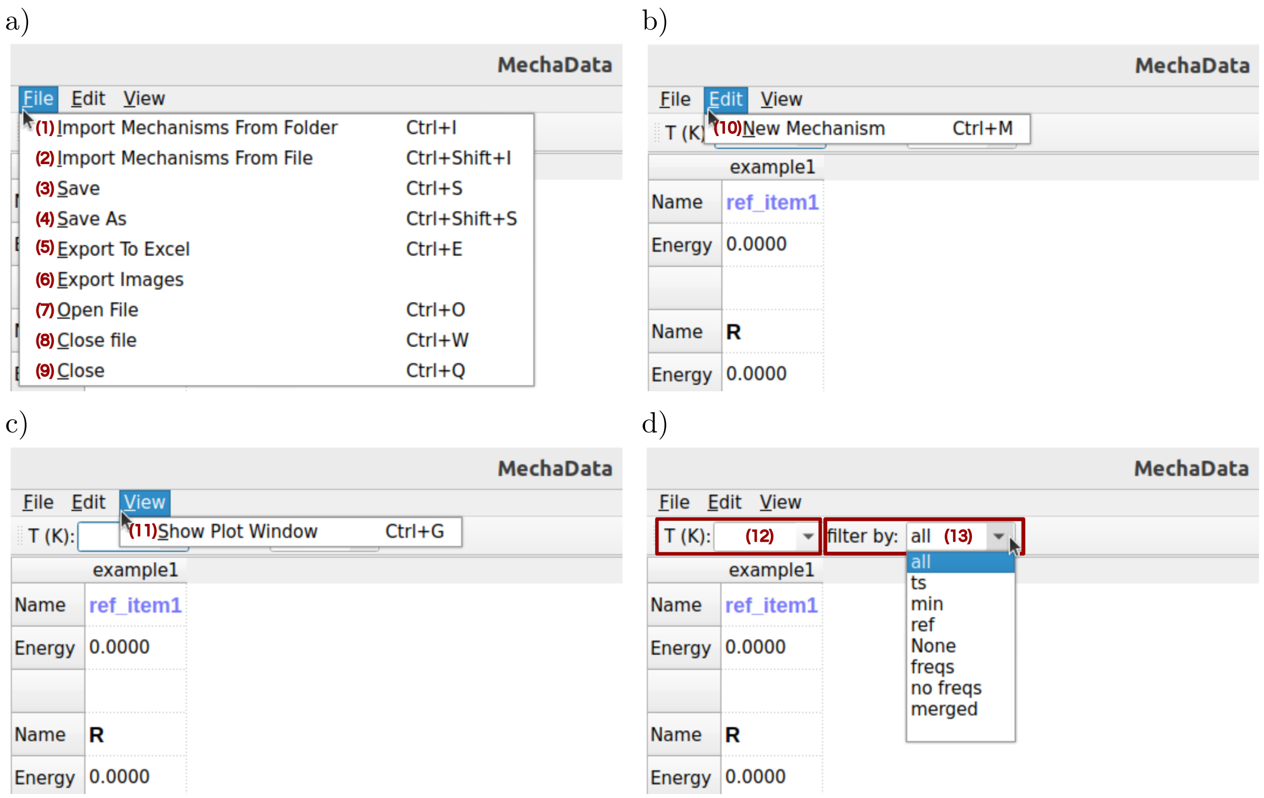

Figure S4: Overview of *MechaData* GUI showing the available menus a–c) and the filtering options d), which allow to filter the calculations by temperature and type.

- **(15) Order Items:** Change the order of the items in a column.
- **(16) Merge Items:** Merges intermediates to simplify the mechanism representation.
- **(17) Import Intermediate:** Adds new species to the mechanism by importing them from external quantum chemical output.
- **(18) Add Reference:** Adds a new energy reference by combining several reference items.
- **(19) Convert Units:** Converts energy units (e.g., Hartree to kJ/mol) across the mechanism for consistency.
- **(20) Change Mechanism Name:** Renames the mechanism column.

- **(21/22) Delete Plot / Delete References:** Removes specific plots or reference entries from the mechanism.

a)

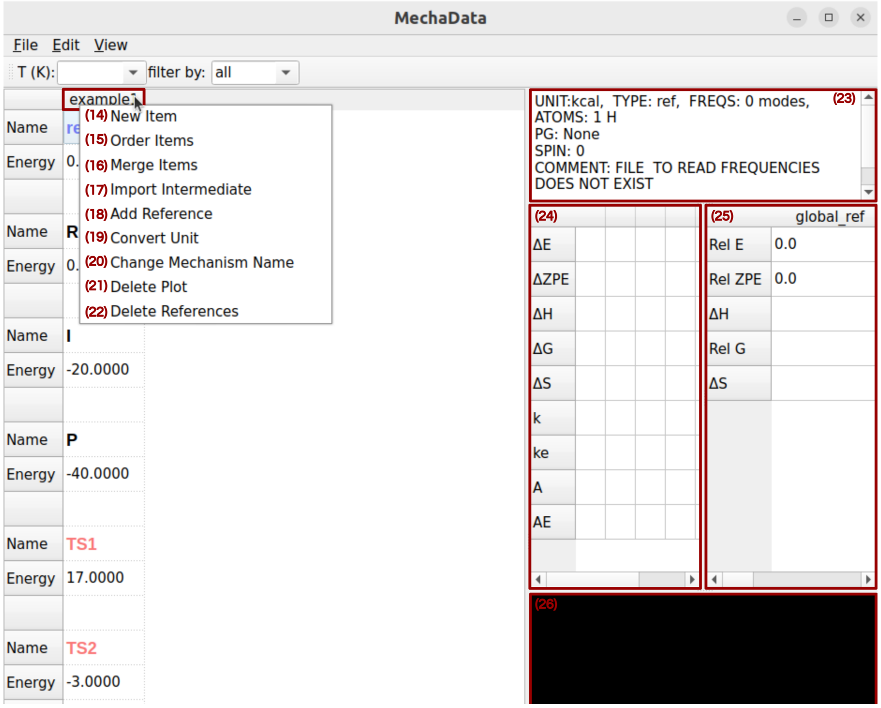

b)

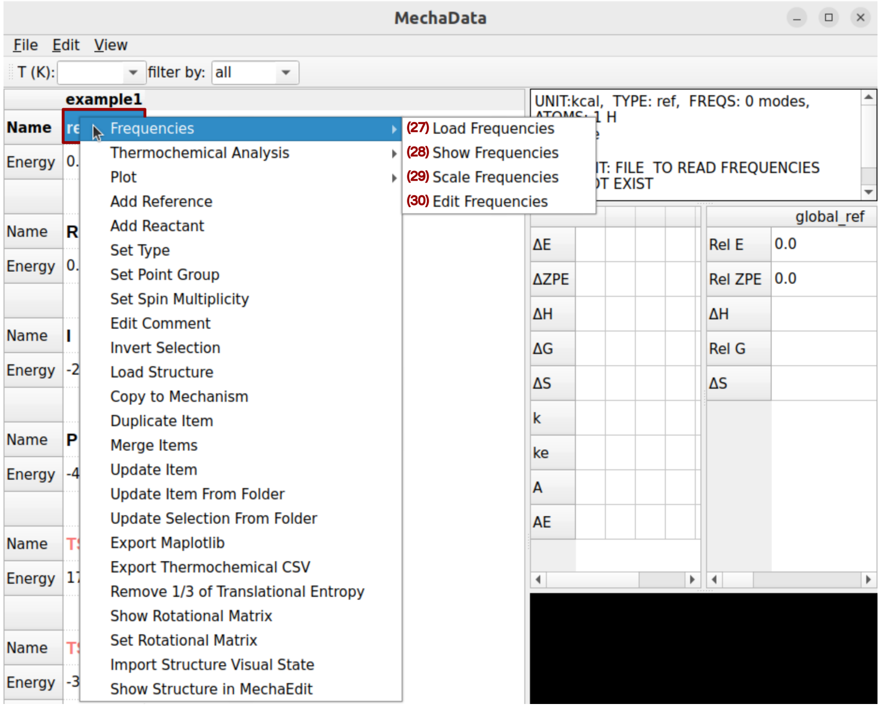

Figure S5: Overview of right-click options for a) mechanism-Level and b-d) species-level controls. In a), the information panel (23), relative energy panel (24), reaction energy panel (25) and embedded visualizer for molecular structures (26) are also highlighted.

c)

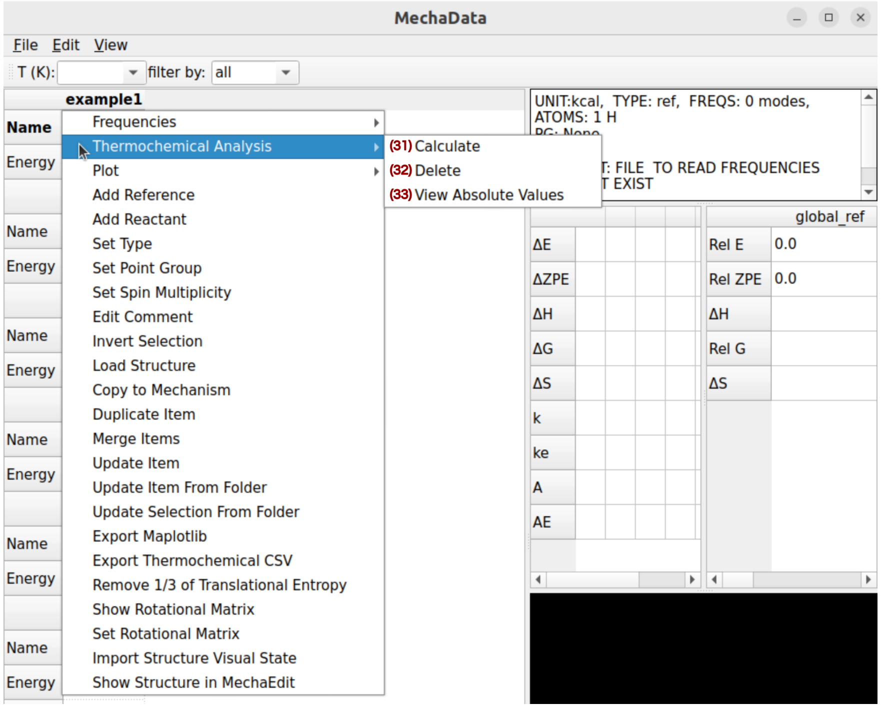

d)

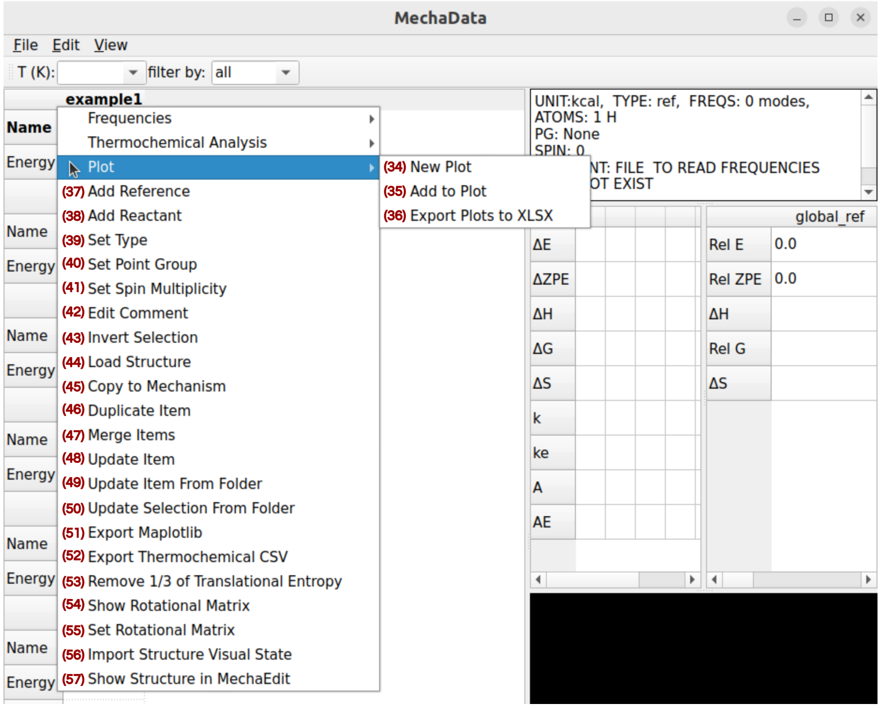

Figure S5: Overview of right-click options for a) mechanism-Level and b-d) species-level controls. In a), the information panel (23), relative energy panel (24), reaction energy panel (25) and embedded visualizer for molecular structures (26) are also highlighted. (*continued*)

S4.1.5 Right-Click Options on Row Entries (Species-Level Controls, Figure S5b-d)

- **(27-30) Frequencies:** Opens a panel to load, view, scale, or edit vibrational frequencies from quantum chemical calculations.
- **(31-33) Thermochemical Analysis:** Computes thermodynamic parameters (G, H, S) based on vibrational and structural data.
- **(34-36) Plot:** Generates energy profiles or add selected intermediates to existing free energy profiles.
- **(37/38) Add Reference / Add Reactant:** Designates the selected species as a reference or adds it as a reactant to the mechanism.
- **(39) Set Type (Minimum, Transition State, Reference):** Categorizes the species for use in kinetics and visualization.
- **(40) Set Point Group:** Assigns a symmetry point group, which may be used in entropy corrections.
- **(41) Set Spin Multiplicity:** Assigns spin multiplicity for electronic entropy.
- **(42) Edit Comment:** Adds or modifies user annotations for the species.
- **(43) Invert Selection:** Toggles current selections. Useful for batch operations.
- **(44) Load Structure:** Imports molecular geometry data from a file.
- **(45) Copy to Mechanism (Column):** Duplicates the selected species into another column.
- **(46) Duplicate Item:** Creates a copy of the current entry.

- **(47) Merge Items:** Create a new intermediate by grouping individual intermediates or transition states. The effect would be to sum up individual energies.
- **(48) Update Item:** Updates the calculation results, energy, geometry, vibrational frequencies, etc., from the calculation folder. The calculation folder must have the same name as the intermediate, and be located in the same working directory.
- **(49) Update Item From Folder:** Updates the calculation results, energy, geometry, vibrational frequencies, etc., from the specified folder.
- **(50) Update Selection From Folder:** Updates the calculation results, energy, geometry, vibrational frequencies, etc., from the specified folder. The folder must contain subfolders matching the names of the selected items.
- **(52) Export Thermochemical CSV:** Saves thermodynamic data for the selected species to a CSV file.
- **(53) Remove 1/3 of Translation Entropy:** Applies a correction often used for adsorbed species in surface chemistry.[S10]
- **(56) Import Structure Visual State:** Modifies, for the selected items, the rotation matrix used to show the geometry in the embedded visualizer.
- **(57) Show Structure in *MechaEdit*:** Opens the species in *MechaEdit*, the advanced structure editor, for detailed modification or inspection.

This structured interface enables users to manage complex quantum chemical data with clarity and flexibility, supporting advanced workflows for thermochemical analysis and kinetic modeling in a research context.

S4.1.6 Calculation of relative energies

In the second example of the manuscript, the first three minima in every column, ethyl bromide, TBOH, and TBAF, are not directly involved in the reaction pathways but are

used as reference species to compute the relative energies of all other intermediates. They form a basis to calculate the relative energies of all intermediates and transition states of a mechanism. A corresponding reference energy can be then calculated as a linear combination of the energies of these reference geometries (Equation S9).

$$\mathrm{E}_{ref} = a\ \mathrm{E}_{CH_3CH_2Br}\ +\ b\ \mathrm{E}_{TBAF}\ +\ c\ \mathrm{E}_{TBOH} \tag{S9}$$

Here, a, b, and c are coefficients automatically determined by *MechaData* through the solution of a system of linear equations. Subsequently, the relative energy of any structure X ($\mathrm{E}_{rel}(X)$, Equation S10) is then computed by subtracting the reference energy (E_{ref}, using Equation S9) to the absolute energy of X ($\mathrm{E}_{abs}(X)$).

$$\mathrm{E}_{rel}(X) = \mathrm{E}_{abs}(X) - \mathrm{E}_{ref} \tag{S10}$$

In this example, we define an intermediate, denoted *REACTANT*, corresponding to the minimum-energy state preceding the *SN2* and *E2* transition states. *REACTANT* is constructed by merging two separate calculations, the geometry optimization of ethyl bromide and the optimization of TBAF microsolvated by 0, 1, 2, or 3 TBOH molecules. Consequently, the absolute energy of *REACTANT* is given by the sum of the energies obtained from these individual optimizations. For *REACTANT* without TBOH, the reference coefficients in Equation S9 are $a = b = 1$ and $c = 0$. These coefficients may vary depending on the atomic composition of each minimum and transition-state structure in the mechanism, ensuring that all energies remain comparable. For example, when *REACTANT* is defined by merging optimized ethyl bromide with optimized TBAF in the presence of one TBOH molecule, the coefficients become $a = b = c = 1$. If two TBOH molecules are present, the coefficient c increases accordingly to 2.

Importantly, the reference coefficients associated with a given intermediate are independent of how the structure is generated, being identical whether the intermediate comes from

a single optimization including all molecules or from merging individually optimized geometries.

By referencing every structure in the mechanism to this common basis and through the appropriate coefficients, all species can be consistently included within the same free-energy profile. This merging and referencing scheme provides a flexible definition of intermediates, avoids unnecessary additional calculations, and removes the need for manual coefficient assignment when computing relative energies. Moreover, because reaction and activation energies are evaluated relative to a common reference basis, overall energy consistency is inherently ensured.

S5 *MechaEdit* module

Although the basic geometry visualization capabilities are integrated into *MechaData*, *MechaEdit* is specifically designed for detailed structural analysis and editing. In addition to the menu bar (Figure S6a), the GUI is composed of a toolbar (Figure S6b), the main drawing canvas (Figure S6c), and a side panel (Figure S6d). The most relevant options are described below.

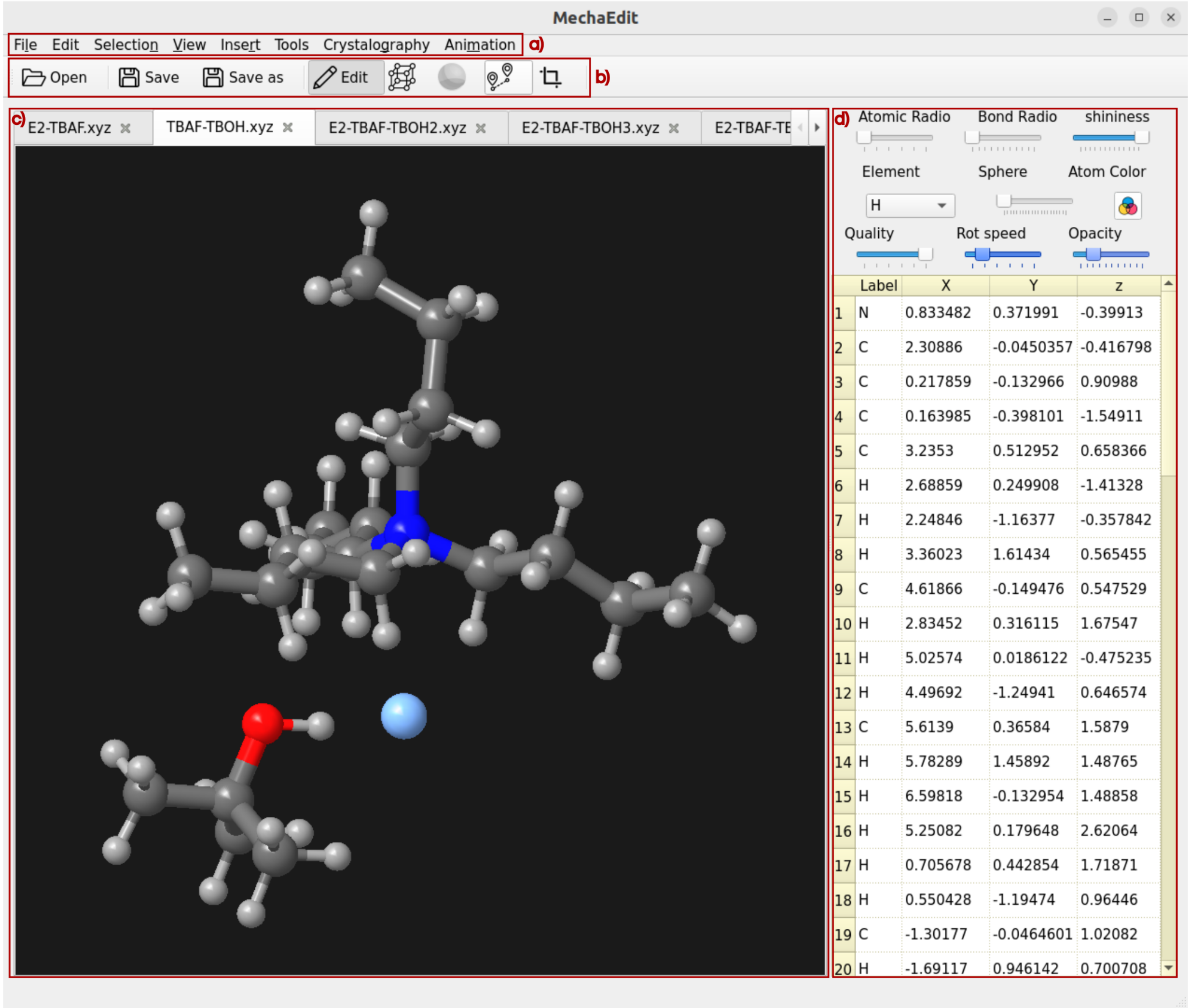

Figure S6: Overview of *MechaEdit* GUI.

S5.1 Toolbar

The following description corresponds to the functionality of the buttons in the Toolbar panel (Figure S6b).

- **Open:** opens a new geometry file.
- **Save:** saves the visualized geometry to the currently opened file with the same name.
- **Save as:** saves the visualized geometry to a different file.
- **Edit:** activates the edit mode, in which atoms that are selected can be dragged (while moving the cursor with the mouse left button clicked) or rotated (while moving the cursor with the mouse right button clicked.
- **Lattice:** toggles the visualization of the unit cell on or off.
- **Specular:** toggles on and off the specular effect on the drawn objects (spheres, cylinders, etc.).
- **Track:** enables track mode for the currently selected atoms. After selecting two atoms sequentially, their interatomic distance is tracked by activating this option. When three atoms are selected sequentially, the distances (in selection order) and the angle are tracked. When four atoms are selected sequentially, the distances (in selection order), angles, and the torsion angle are tracked. After the track mode is enabled, the selection can be modified and any change made with the edit mode is continuously tracked, and their values (distances, angles, and torsion angles) are updated in the bottom bar of *MechaEdit* window.
- **Crop:** activates the crop mode, in which a rectangular region of the drawing canvas can be defined before image rendering and export. It is useful for controlling the size and which part of the structure will be included in the saved image.

S5.2 Drawing canvas

In the drawing canvas (Figure S6c), the structure can be rotated by holding the left mouse button and moving the mouse. The view can be translated by holding the right mouse button and moving the mouse. Individual atoms can be selected using Ctrl + left click, while a range of atoms can be selected by holding Ctrl and dragging with the left mouse button. Selected atoms can be deleted using the Delete key. Selected atoms can be hidden with Ctrl + H shortcut.

S5.3 Side panel

The side panel (Figure S6d) contains a set of slide bars and buttons designed to intuitively modify the properties of the canvas elements (atomic properties, visual properties) and canvas behavior). Besides, the included coordinates editor allows selecting and modifying the atomic coordinates individually. In addition, a frequency and frames listbox can be added to the side panel. The frequency listbox can be enabled/disabled from the tool menu. The frames listbox is automatically enabled if the opened file is a trajectory.

S5.4 Menu Options

S5.4.1 File Menu

The File menu contains options for loading, saving, and exporting chemical structures.

- **New:** creates a new, empty structure.
- **Open:** loads a structure file into the visualization canvas.
- **Save:** saves the current structure to the active file.
- **Save As:** saves the current structure to a new file location.

- **Render (POV-Ray):** exports the current structure for high-quality rendering using POV-Ray, suitable for publication-quality images.
- **Close:** closes the program.

S5.4.2 Edit Menu

The Edit menu provides basic editing operations for selected atoms.

- **Copy:** copies the currently selected atoms to the clipboard.
- **Paste:** inserts previously copied atoms into the structure.
- **Center:** centers the structure in the drawing canvas.

S5.4.3 Selection Menu

The Selection menu provides tools to create and modify atom selections.

- **Select all:** selects all atoms in the current structure.
- **Invert selection:** reverses the current selection state.
- **Select by bonds:** expands the selection based on bonding connectivity.
- **Select inside sphere:** selects all atoms inside a given radius from a previously selected atom.
- **Select groups:** selects previously created groups (in tools menu).

S5.4.4 View Menu

The View menu controls the representation of the structure.

- **Restore defaults:** changes the sphere and cylinder radii, and colors to the predefined values.

- **Models:** allows changing to a licorice or wire representation.
- **Atom:** allow changing atomic properties and representation.
- **Background color:** changes the background color of the canvas.
- **Styles:** allows changing predefined styles from files.
- **Set zoom:** allows defining a zoom ratio with respect to the default zoom to control how far the structure is visualized.

S5.4.5 Insert Menu

The Insert menu allows the insertion of structures in the current representation.

- **Fragment:** inserts a structure from a file into the current visualization.
- **Download IZA:** downloads a zeolite CIF file from the IZA website (`https://europe.iza-structure.org/IZA-SC/ftc_table.php`).

S5.4.6 Tools Menu

The Tools menu provides interactive analysis and measurement features.

- **Fix Selected:** sets a fixed flag internally to *true* so that the saved structure in VASP (POSCAR) format contains the selective dynamics flags.
- **Unfix Selected:** sets a fixed flag internally to *false* so that the saved structure in VASP (POSCAR) format contains the selective dynamics flags.
- **Translate:** allows translating the selected atoms by adding an input vector, or to specified x, y, or z positions.
- **Rotate:** rotates the whole structure around the z axis or bonds (by preselecting the two atoms of the bond).

- **Repeat:** replicates the unit cell along the x, y and/or z direction by the specified amount. There must be a unit cell defined in the structure.

- **Coordinates:** shows or hides the coordinates listbox in the side panel.

- **Create group:** with this option, atoms can be grouped for future selection and manipulations.

S5.4.7 Crystallography

- **Set Unit Cell:** opens a dialog to define or modify unit cell parameters.

S5.4.8 Animation Menu

The Animation menu allows playback of dynamic structural data.

- **Play:** plays an available animation or trajectory.

- **Play Frequencies:** animates vibrational modes, typically obtained from frequency calculations.

- **Time Delay:** adjusts the delay between animation frames to control playback speed.

S5.4.9 Notes

Most menu actions operate on the currently selected atoms. Atom selection is performed directly in the drawing canvas using mouse and keyboard controls. Available visualization, animation, and analysis features depend on the data present in the loaded structure.

S6 *MechaKinetics* module

The *MechaKinetics* module accepts input data in JSON format, which defines the chemical reactions, temperature-dependent rate data, simulation time parameters, and initial species concentrations. The input file can be generated in the *MechaData* plotting interface (right-click and *export reaction network*) and customized as needed. As shown in Figure S7, it has the following sections:

```
{
    "mec": {
        "A+B=I": {
            "298": [
                2.1757017831042536,
                4.832099086983306e-15
            ]
        },
        "I=P": {
            "298": [
                2.1757017831042536,
                4.832099086983306e-15
            ]
        }
    },
    "time": "0 8 1000",
    "initial_values": {
        "A": 1,
        "B": 1
    }
}
```

Figure S7: Input structure of JSON input file for *mechakinetics.py*

- **Reaction Data:** The `"mec"` field contains the chemical reactions and their kinetic parameters. Each key corresponds to a reaction, that is written in the general format `"aA+bB=cC+dD"`. Within each reaction, the rates are provided as a dictionary of temperatures (in Kelvin) and a list containing two numbers, e.g: `"298": [2.17, 4.83e-15]`. The first number is the forward reaction rate and the second the reverse reaction rate (both in s^{-1}), calculated using Eyring's equation. Multiple temperatures can be included if the reaction rate needs to be evaluated across a temperature range.

- **Simulation Time:** The `"time"` field defines the time span (in seconds) for the kinetics

simulation as a string of three values: `"time": "t_start t_end t_step"`.

- **Initial Values:** The `"initial_values"` field defines the starting concentrations of reacting species, for example, `"initial_values": { "A": 1, "B": 1 }`. Here, A and B start with a concentration of 1 (in arbitrary units consistent across all species). All other species are assumed to start at zero unless explicitly specified.

The user is encouraged to see other examples of these input files in the examples folder of the source code, such as *rn.json*, and *reaction_network_0tboh.json*.

S7 Example: General workflow in *MechaSuite*

This example illustrates the general environment of *MechaData*. The hypothetical reaction is a simple two-step mechanism occurring in a batch reactor, where the concentration of reactants changes over time until reaching an equilibrium. The reaction network is defined as follows:

$$\mathrm{R} \underset{k_{1,r}}{\overset{k_{1,f}}{\rightleftharpoons}} \mathrm{I} \tag{R3}$$

$$\mathrm{I} \underset{k_{2,r}}{\overset{k_{2,f}}{\rightleftharpoons}} \mathrm{P} \tag{R4}$$

where R, I and P are the hypothetical reactant, intermediate and product, respectively. The rate constants for both steps are set to be equal by assigning identical forward Gibbs free energies of activation, such as $\Delta G^{\ddagger}_{1,f} = \Delta G^{\ddagger}_{2,f} = 17$ kcal/mol. Using Eyring's equation, the corresponding forward rate constants at 298 K are $k_{1,f} = k_{2,f} = 2.17$ s^{-1}. To make the steps irreversible, the reverse constants are deliberately chosen to be small, $k_{1,r} = k_{2,r} \sim 10^{-15}$ s^{-1}. This can be achieved by setting the $\Delta G^{\ddagger}$ for the reverse steps considerably higher than the forward ones, like $\Delta G^{\ddagger}_{1,r} = \Delta G_{2,r} = 57$ kcal/mol.

Figure S8a illustrates the main interface of *MechaData*, displaying the central spreadsheet that lists the hypothetical minima species (R, I, and P) together with the corresponding

transition states ($TS1$ and $TS2$) and their associated hypothetical energies. Their relative energies with respect to the global reference (denoted as *global_ref*), along with the corresponding activation and reaction energies, are displayed in the smaller spreadsheets on the right (relative energy and reaction energy panels in Figure S3b,c). In this example, the *global_ref* serves merely as an arbitrary zero-energy reference for illustration.

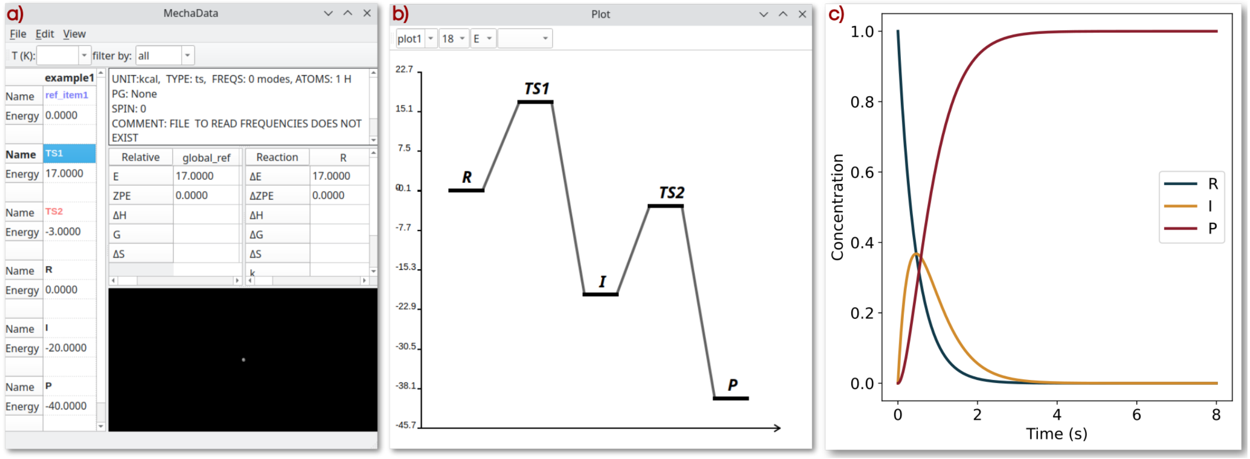

Figure S8: *MechaData* interface. a) Main spreadsheet with absolute and relative energies for species R, I and P as well as for $TS1$ and $TS2$. b) Plotting tool for customizing graph appearance prior to export. c) Time evolution of the concentration of R, I and P.

The interactive plotting interface displaying the corresponding energy profile derived from the reaction mechanism is shown in Figure S8b. The graphical elements representing minima and transition states, as well as their labels, can be customized in terms of style, color, line width, and position. Once the desired settings have been defined, a publication-ready version of the plot can be generated using Matplotlib (see the second example in the manuscript).

The concentration profiles in Figure S8c were obtained by numerically solving the system of differential equations using *MechaKinetics*. The results illustrate that the concentration of R decreases over time, the intermediate I first increases and then declines after approximately 0.1 seconds, and the product P increases exponentially, behavior characteristic of a first-order reaction with respect to R.

Beyond their illustrative role, this and many other hypothetical examples provide a valuable framework for educational purposes. By systematically varying kinetic and thermody-

namic parameters, users can explore how individual elementary steps influence the overall behavior of a reaction network. Such interactive exploration facilitates an intuitive understanding of reaction kinetics, sensitivity to model parameters, and the interplay between mechanism and observable rates, making these examples particularly well suited for teaching and training in microkinetic modeling.

References

(S1) Chorkendorff, I.; Niemantsverdriet, J. W. *Concepts of Modern Catalysis and Kinetics, 3rd Edition*; Wiley: Weinheim, Germany, 2017.

(S2) Espenson, J. H. *Chemical Kinetics and Reaction Mechanisms*; McGraw-Hill: Maidenhead, England, UK, 1981.

(S3) Cramer, C. J. *Essentials of Computational Chemistry: Theories and Models*; John Wiley & Sons: Hoboken, NJ, USA, 2013.

(S4) Eyring, H. The Activated Complex in Chemical Reactions. *J. Chem. Phys.* **1935**, *3*, 107–115.

(S5) Evans, M. G.; Polanyi, M. Some applications of the transition state method to the calculation of reaction velocities, especially in solution. *Trans. Faraday Soc.* **1935**, *31*, 875–894.

(S6) Eyring, H.; Polanyi, M. On Simple Gas Reactions. *Z. Phys. Chem.* **1931**, *227*, 1221–1246.

(S7) Motagamwala, A. H.; Dumesic, J. A. Microkinetic Modeling: A Tool for Rational Catalyst Design. *Chem. Rev.* **2021**, *121*, 1049–1076.

(S8) Inc., T. M. MATLAB version: 9.13.0 (R2022b). 2022; https://www.mathworks.com.

(S9) Medasani, B.; Kasiraju, S.; Vlachos, D. G. OpenMKM: An Open-Source C++ Multiscale Modeling Simulator for Homogeneous and Heterogeneous Catalytic Reactions. *J. Chem. Inf. Model.* **2023**, *63*, 3377–3391.

(S10) Rzepa, C.; Siderius, D. W.; Hatch, H. W.; Shen, V. K.; Rangarajan, S.; Mittal, J. Computational Investigation of Correlations in Adsorbate Entropy for Pure-Silica Zeolite Adsorbents. *J. Phys. Chem. C* **2020**, *124*, 16350–16361.
